# Sex-specific autophagy modulation in osteoblastic lineage: a critical function to counteract bone loss in female

**DOI:** 10.18632/oncotarget.12013

**Published:** 2016-09-13

**Authors:** Olivier Camuzard, Sabine Santucci-Darmanin, Véronique Breuil, Chantal Cros, Tatiana Gritsaenko, Sophie Pagnotta, Laurence Cailleteau, Séverine Battaglia, Patricia Panaïa-Ferrari, Dominique Heymann, Georges F. Carle, Valérie Pierrefite-Carle

**Affiliations:** ^1^ UMR E-4320 TIRO-MATOs CEA/DRF/BIAM, Université Nice Sophia Antipolis, Faculté de Médecine Nice, Nice, France; ^2^ Service de Chirurgie Réparatrice et de la Main, CHU de Nice, Nice, France; ^3^ Service de Rhumatologie, CHU de Nice, Nice, France; ^4^ Centre Commun de Microscopie Appliquee, Université Nice Sophia Antipolis, Nice, France; ^5^ Plateforme Imagerie IRCAN, Faculté de Médecine, Université Nice Sophia Antipolis, Nice, France; ^6^ INSERM UMR 957 Université de Nantes, Equipe labellisée Ligue Nationale Contre le Cancer, Nantes, France; ^7^ Laboratoire de Biochimie, CHU de Nice, Nice, France; ^8^ Department of Oncology and Metabolism, The Medical School, University of Sheffield, Sheffield, UK

**Keywords:** osteoblast, osteocyte, autophagy, osteoporosis, aging, Gerotarget

## Abstract

Age-related bone loss is associated with an increased oxidative stress which is worsened by estrogen fall during menauposis. This observation has drawn attention to autophagy, a major cellular catabolic process, able to alleviate oxidative stress in osteoblasts (OB) and osteocytes (OST), two key bone cell types. Moreover, an autophagy decline can be associated with aging, suggesting that an age-related autophagy deficiency in OB and/or OST could contribute to skeletal aging and osteoporosis onset.

In the present work, autophagy activity was analyzed in OST and OB in male and female mice according to their age and hormonal status. In OST, autophagy decreases with aging in both sexes. In OB, although a 95% decrease in autophagy is observed in OB derived from old females, this activity remains unchanged in males. In addition, while ovariectomy has no effect on OB autophagy levels, orchidectomy appears to stimulate this process. An inverse correlation between autophagy and the oxidative stress level was observed in OB derived from males or females. Finally, using OB-specific autophagy-deficient mice, we showed that autophagy deficiency aggravates the bone loss associated with aging and estrogen deprivation.

Taken together, our data indicate that autophagic modulation in bone cells differs according to sex and cell type. The lowering of autophagy in female OB, which is associated with an increased oxidative stress, could play a role in osteoporosis pathophysiology and suggests that autophagy could be a new therapeutic target for osteoporosis in women.

## INTRODUCTION

The coordinated action of osteoblasts (OB), the cells responsible for bone formation, and osteoclasts (OC), the cells specialized for bone resorption, ensures bone remodeling which is under the control of osteocytes (OST), the multifunctional mechanosensing cells embedded in the bone matrix [1]. The remodeling process is highly active throughout life and perturbation of this process can lead to many bone metabolic defects including osteoporosis (OP). This pathology, due to an imbalance favoring bone resorption over formation, is characterized by increased OB apoptosis as well as an enhanced OC number and activity, leading to increased bone fragility and susceptibility to fracture [2, 3]. Currently, one in three women and one in five men over 50 year-old will expe­rience an osteoporotic fracture [3]. At the molecular level, age-related bone loss is associated with an increased oxidative stress that is worsened by estrogen fall during menauposis in women [4, 5]. This observation has drawn attention to autophagy, a major degradation pathway able to limit oxidative stress through the elimination of dysfunctional mitochondria [6, 7]. During this process, the cytoplasmic material targeted to degradation is sequestered within double-membraned vesicles called autophagosomes which then fuse with lysosomes. Autophagic degradation products are finally delivered back to the cytoplasm for recycling or energy production [6, 8]. Autophagy occurs at low level in all cells to ensure the homeostatic turnover of long-lived proteins and organelles [9] and is upregulated under stressfull conditions [8]. In bone, autophagy appears to be essential for bone homeostasis [10–14] and a link between autophagy genes and osteoporosis has been highlighted in human genome-wide association data [15]. It is generally accepted that autophagy declines with age [16–20] and an age-related autophagy deficiency in OB and/or OST could thus contribute to skeletal aging and OP onset. A decreased activity of OST autophagy with aging has been observed in male rats [21]. In addition, ovariectomy was shown to increase autophagy in female rat OST [22]. In the present work, we have analyzed autophagic activity in OST and OB in male and female mice according to their age and hormonal status. Moreover, we have used OB-specific autophagy-deficient mice to analyze how autophagy defect can affect bone mass in old ovariectomized mice.

## RESULTS

### Autophagic activity in osteocytes during aging or gonadectomy in male and female mice

**Figure 1:**
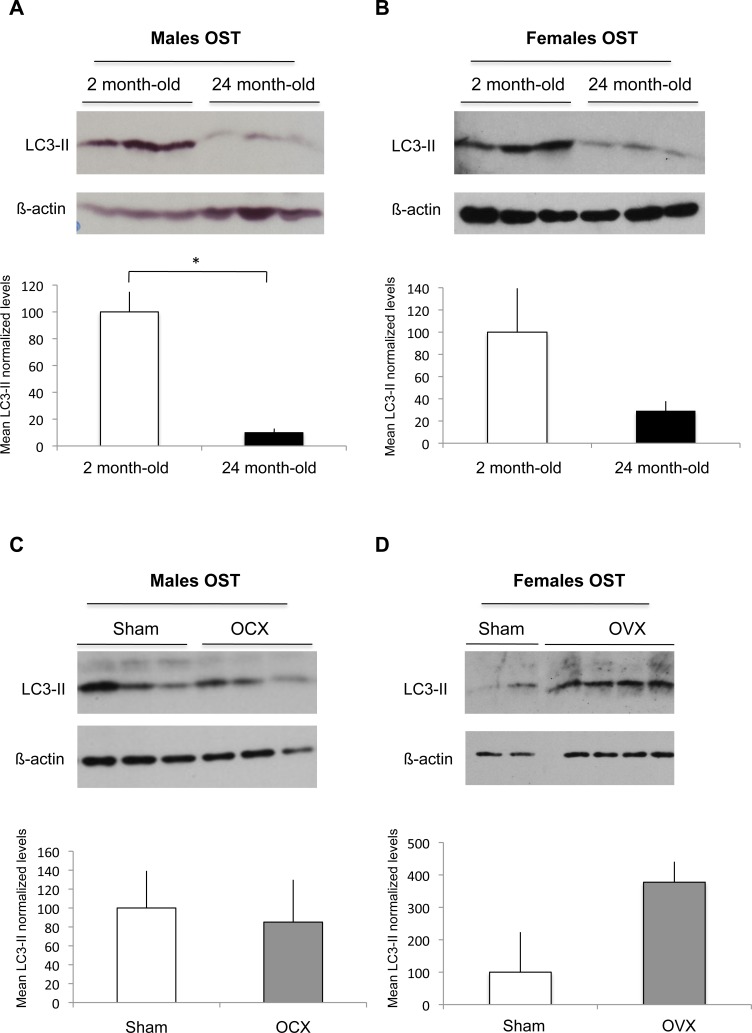
Effect of aging and gonadectomy on autophagosome number in OST LC3-II level, which reflects autophagosome number, was determined by Western blot in cortical bone. LC3-II to β-actin relative levels are expressed as means ± standard errors. **A.** Comparison of LC3-II expression in cortical bone from 2 month-old (*n* = 3) and 24 month-old (*n* = 3) male mice. **B.** Comparison of LC3-II expression in cortical bone from 2 month-old (*n* = 3) and 24 month-old (*n* = 3) female mice. **C.** Comparison of LC3-II expression in cortical bone from sham (*n* = 3) and OCX (*n* = 3) male mice. **D.** Comparison of LC3-II expression in cortical bone from sham (*n* = 2) and OVX (*n* = 4) female mice. * = *P* < 0.05.

As osteocytes compose 90% to 95% of all bone cells in adult skeleton [23], we analyzed autophagic activity of OST by using cortical bone which represent an OST-enriched tissue. Upon autophagy induction, the essential autophagy protein microtubule-associated protein 1 light chain 3 protein (LC3-I) becomes lipidated (LC3-II) and inserts into the autophagosome membrane [24]. One of the widely used methods to detect autophagy is thus based on the quantification of the LC3-II protein by western blot. As shown in Figure 1A, we observed a 90% decrease in the steady-state levels of the LC3-II protein in the 24 month-old male mice compared to the 2 month-old mice, suggesting a decrease in the number of autophagosomes. Similarly, a 70% decrease in LC3-II protein levels was observed in old female mice compared to the young, suggesting that aging induces an autophagy decline in OST both in male and female mice (Figure 1B). We next assessed autophagy in OST of male and female mice after orchidectomy (OCX) or ovariectomy (OVX), respectively. Although OCX had no effect on OST autophagy (Figure 1C), our results suggest that OVX induced an increase in OST autophagic activity (Figure 1D).

### Autophagic activity in osteoblast during aging in male and female mice

**Figure 2:**
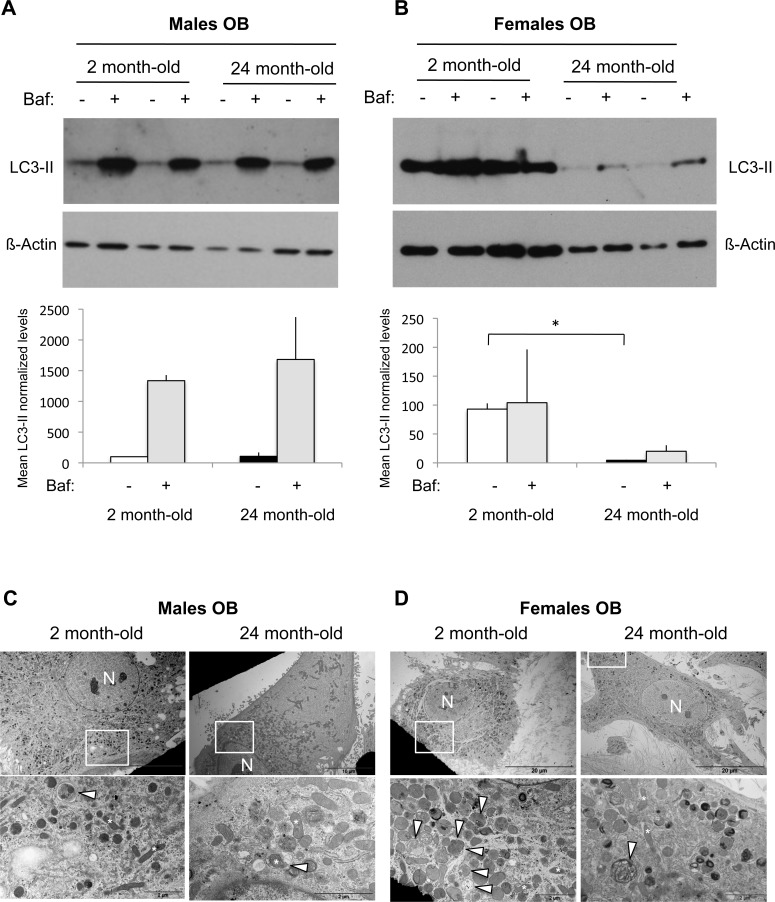
Autophagic activity in OB from young and old male and female mice OB cells were isolated from long bones of 2 month-old (*n* = 2) and 24 month-old (*n* = 2) male and female mice. **A.-B.** LC3-II expression analysis by Western blot. Two hours prior to protein extraction, Bafilomycin A1 (Baf) was added or not to block to the autophagic flux. LC3-II to β-actin relative levels are expressed as means ± standard errors. **C.-D.** Analysis of autophagic vacuoles by transmission electron microscopy. The boxed areas are shown at higher magnification. arrowhead: autophagic vesicle, N: nucleus. * = *P* < 0.05.

As we recently demonstrated that autophagy in OB is a key player in mineralization and bone homeostasis [11], we next assessed autophagy in OB isolated from long bones of young and old mice. Regarding OB isolated from male mice, no difference was observed in LC3-II levels between 2 month-old and 24 month-old mice (Figure 2A). Autophagy is a dynamic mechanism in which vesicles are formed and rapidly degraded by fusion with the lysosome, in a process called “autophagic flux”. The simple determination of numbers of autophagosomes (ie LC3-II level) is insufficient for an overall estimation of autophagic activity and a current method used to measure autophagic flux is the monitoring of LC3 turnover [24, 25]. Thus, we used the lysosomal proton pump inhibitor Bafilomycin-A1 to clamp the LC3-II autophagosome degradation. As shown Figure 2A, this resulted in a dramatic increase in the LC3-II signal whatever the age of the animals, indicating that the autophagic flux is robust. In the case of OB isolated from female mice, we observed a 95% decrease of the LC3-II levels in old compared to the young mice, suggesting a reduced autophagosome number (Figure 2B). Addition of Bafilomycin-A1 induced a very slight increase in the LC3-II signal, indicating that the autophagic flux is less robust than in males. TEM analysis allowed to confirm these data as shown in Figure 2C and 2D. Although the number of autophagic vesicles was not significantly different in OB from young and old male mice, aging was associated with a decreased number of autophagic vacuoles in females.

### Autophagic activity in osteoblasts after gonadectomy in male and female mice

**Figure 3:**
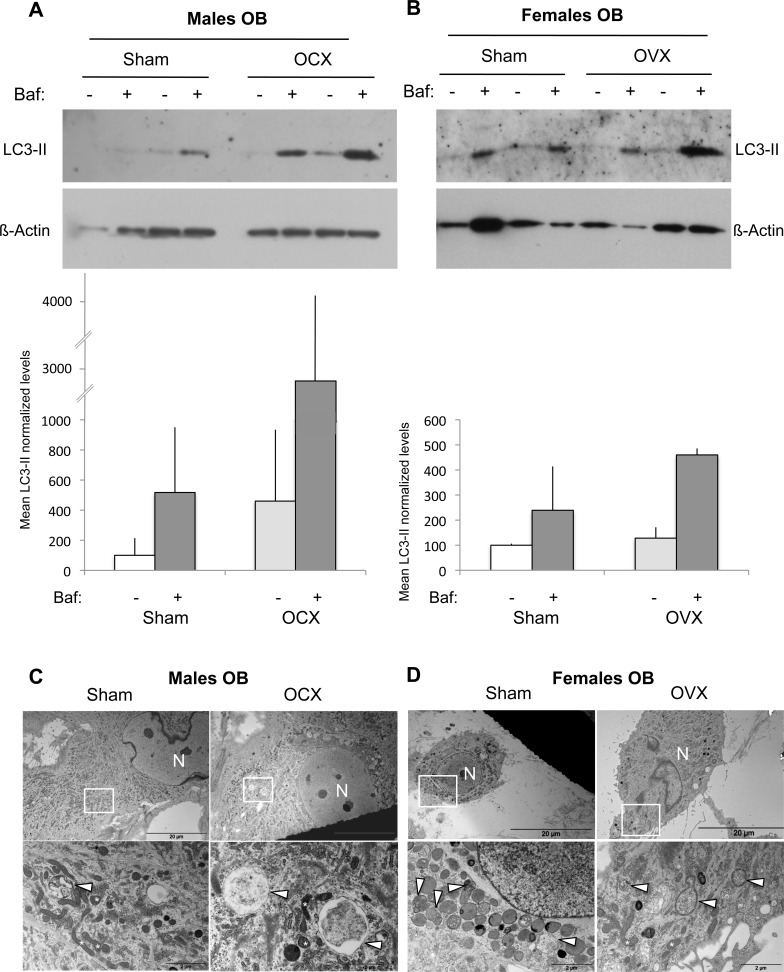
Autophagic activity in OB from gonadectomized mice OB cells were isolated from long bones of male (*n* = 2) and female (*n* = 2) mice two months after OCX and OVX, respectively. **A.-B.** LC3-II expression analysis by Western blot. Two hours prior to protein extraction, Bafilomycin A1 (Baf) was added or not to block to the autophagic flux. LC3-II to β-actin relative levels are expressed as means ± standard errors. **C.-D.** Analysis of autophagic vacuoles by transmission electron microscopy. The boxed areas are shown at higher magnification. arrowhead: autophagic vesicle, N: nucleus.

We next analyzed the autophagic activity in OB isolated from orchidectomized (OCX) and ovariectomized (OVX) mice. Regarding male mice, we observed an increase tendency in the LC3-II levels following OCX (Figure 3A). This signal was further enhanced in the presence of Bafilomycin-A1, suggesting that OCX was associated with an autophagy induction. In the case of female mice, no difference was observed in the LC3-II signal, indicating that OVX has no influence on autophagy (Figure 3B). These data were confirmed by TEM analysis showing an increased number of autophagic vesicles in OB from OCX mice compared to sham (Figure 3C), while no difference was noted between OB from OVX and sham mice (Figure 3D).

### Aging and gonadectomy influence on oxidative stress in OB

**Figure 4:**
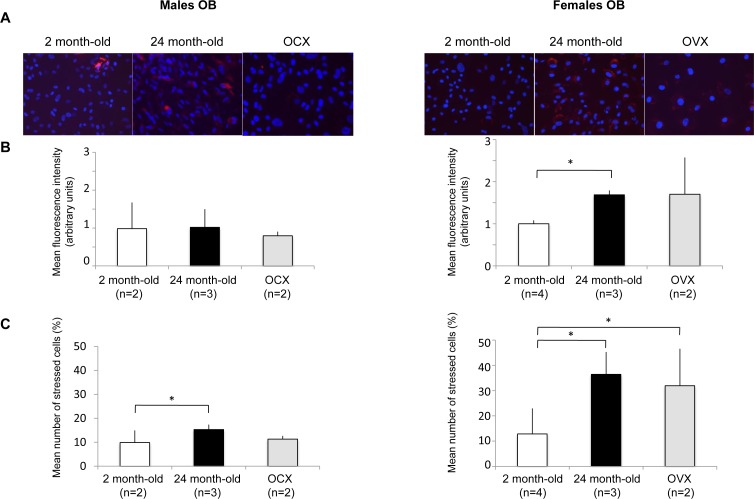
Effect of aging and gonadectomy on oxidative stress in OB OB cells were isolated from long bones of 2 month-old (*n* = 2), gonadectomized (*n* =) and 24 month-old (*n* = 2) male and female mice (2-4 mice per condition). The cells were incubated in the presence of a fluorogenic probe sentitive to the oxidative stress and analyzed by fluorescent microscopy. **A.** A representative picture of each condition is presented. The results are expressed as **B.** the mean fluorescence intensity (arbitrary units) and **C.** the mean number of stressed cells (%) in each condition. * = *P* < 0.05.

Increased levels of oxidative stress in OB has been identified as one of the critical component of bone loss physiopathology [5, 26–28]. As oxidative stress and autophagy are closely related, we then assessed ROS levels in OB from young, old and gonadectomized animals (Figure 4). Two parameters have been analyzed, the stress intensity (Figure 4B) and the percentage of stressed cells (Figure 4C). In males, the stress intensity was not modulated by the experimental conditions. However, a significant increase in the number of stressed cells was observed for OB from old mice. Regarding females, the stress intensity was significantly increased in OB from old mice. A similar increase was observed in OB from OVX mice although not statistically significant due to a greater value variation between individuals. In addition, the number of stressed cells was significantly increased in old and OVX mice. It is interesting to note that both the stress intensity and the number of stressed cells were higher in OB from female than from male mice. These data are further supported by the analysis of mitochondria by transmission electron microscopy (Figure S1). In male OB, mitochondria exhibit a normal morphology whatever the condition, suggesting a modest oxidative stress modulation. Although mitochondria from young female OB exhibit a normal morphology, swollen mitochondria can be observed in old and OVX female OB, consistent with a drastic oxidative stress increase in these conditions [29].

### Consequence of ovariectomy in a mouse model of autophagy deficiency in OB

**Figure 5:**
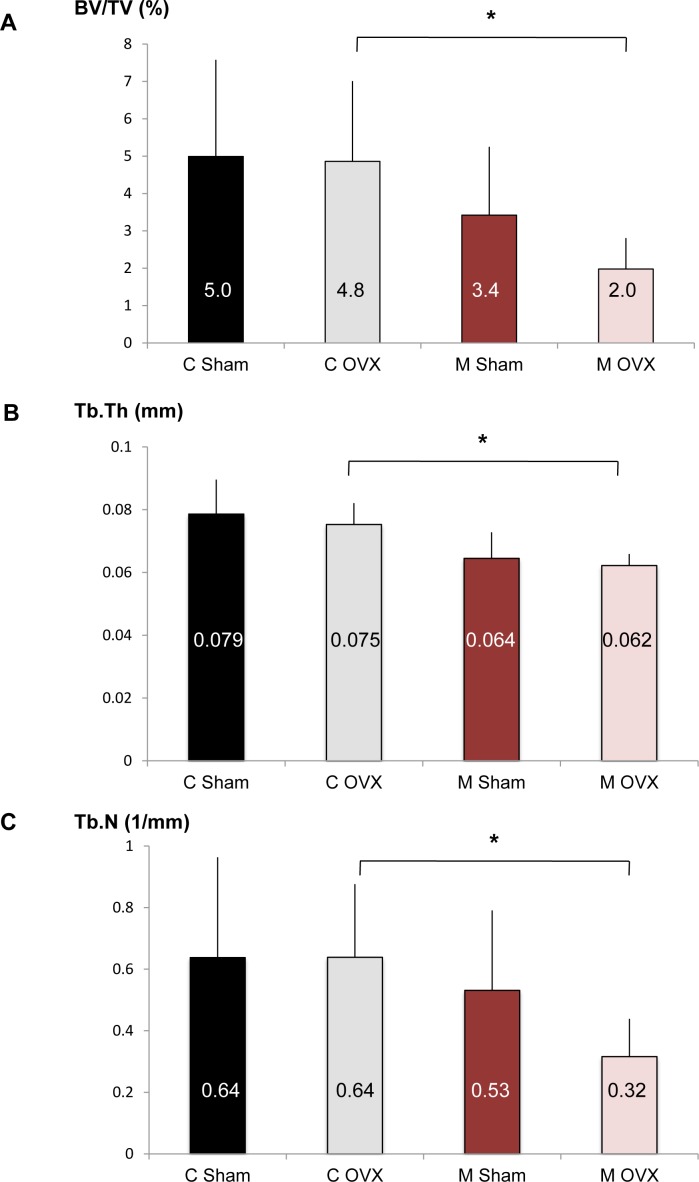
Effect of autophagy deficiency in OB on bone phenotype induced by aging and ovariectomy Eighteen month-old female Atg5Col1A-Cre+ (mutant, M) mice and their control littermates (control, C) were ovariectomized (C OVX, *n* = 6; M OVX, *n* = 4) or sham-operated (C Sham, *n* = 12; M Sham, *n* = 8). **A.-C.** The percentage of bone volume per total volume (BV/TV), the trabecular thickness (Tb.Th) and the trabecular number (Tb.N) in each group are presented. The mean is indicated in each histogram. * = *P* < 0.05.

The above results suggest that autophagy modulation in OB according to age and hormonal status is less favorable in females than in males. To determine whether OB autophagy is a critical function in female to counteract age-related bone loss in the context of estrogen fall, we used OB-specific autophagy-deficient mice generated by breeding of Atg5*^flox-flox^* mice to those expressing Cre recombinase under the control of the osteoblastic type 1a collagen (Col1A) promoter [11]. An ovariectomy was performed in 18 month-old Atg5*^flox-flox^* Col1A-Cre- (control) and Atg5*^flox-flox^* Col1A-Cre-+ (mutant) mice and the skeletal phenotype of these animals was analyzed by microcomputerized tomography (Figure 5). Although OVX doesn't affect the BV/TV ratio in old control mice (C Sham *vs* C OVX), estrogen deprivation lead to a 40% decrease in BV/TV in OB-specific autophagy-deficient mice (M Sham *vs* M OVX). In OVX animals (C OVX *vs* M OVX), autophagy deficiency in OB induced a 60% decrease in bone volume (BV/TV) and significantly reduced trabecular thickness (Tb.Th) and number (Tb.N).

## DISCUSSION

Our results indicate that aging is associated with a reduced number of autophagosomes in OST from male and female mice, suggesting a decreased autophagic activity with aging. A study of Chen et al. previously showed a reduced autophagy in OST from old *vs* young male rats [21]. Our results confirm these data and suggest that a similar decrease is observed in females. Although several studies reported that aging was associated with autophagy decline [16–20], the underlying mechanisms for autophagy reduction in aging tissues are poorly defined. It has been shown that the expression of some autophagy genes was downregulated with aging [20]. In addition to transcriptional regulation, expression of autophagy genes can be regulated by several epigenetic mechanisms, such as chromatin modulation, histone modification, and microRNAs, which can be modified during aging [30].

Regarding autophagy in OB, although aging has no effect in males, it was associated with a reduced autophagic activity in females. A similar age-associated regulation has been observed in skeletal muscle, with a decreased LC3-II level in females and no modification in males [31]. As a consequence, the oxidative stress level is higher in OB from old female than from old male. A study of Almeida et al. indicated that ROS level in bone marrow aspirates from old mice was higher in females than in males [4]. This was correlated with a more rapid age-associated drop in glutathione reductase activity in females [4]. Our results confirm these data in OB and suggest that autophagy decline in females participate in the oxidative stress increase during aging.

In the case of gonadectomy, we observed that OCX had no effect on OST autophagy whereas OVX seemed to increased the LC3-II levels. OVX was also shown to increase autophagic activity in female rat osteocytes [22]. These data suggest that estrogen could negatively regulate autophagy in OST. Autophagy inhibition by estrogen has also been observed in mouse uterus, rat cardiomyocytes and rat motor neurons [32–34]. Conversely, estradiol was shown to increase autophagy mediated by serum deprivation in OB, suggesting that the pro- or anti-autophagy effects of estrogens are cell- and context-dependent [35].

We next analyzed the effects of gonadectomy on OB autophagy and the results suggested that OCX could increase autophagy in male OB. This could be consistent with data obtained in skeletal muscle showing that testosterone deprivation can inhibit mTOR signaling and stimulate autophagy [36, 37]. Consequently, no significant modification in oxidative stress was observed in OB from OCX mice. In females, autophagic activity was not modulated by OVX and oxidative stress was increased, likely due to the loss of antioxidant actions exert by estrogens [5].

These data clearly show that autophagy regulation is different according to cell sex. In addition to exposure to sex hormones, differences observed between male and female cells can result from the genes expressed on sex chromosomes. As sex chromosomes account for 5% of the total human genome [38–40], 1:20 proteins could theorically differ between males and females, which should influence at least some aspects of cellular biochemistry and physiology. For example, it was shown that primary vascular smooth muscle cells exhibit a different basal redox state and a different susceptibility to oxidative stress according to their sex, male cells being more apoptotic-prone and female cells being more senescence-prone in the same experimental conditions [41, 42]. Interestingly, mechanism underlying this difference integrates a higher anoikis resistance in female cells, that can be due, at least in part to a higher propensity to undergo autophagy [43]. Although sex differences at the level of autophagy are observed in many diseases, little is known about the mechanisms underlying this phenomenon [44]. Using bioinformatics resource for studying autophagy regulation, Türei et al. indicated that 84% of the core autophagy proteins are transcriptionally regulated by sex steroid receptors [45]. In the bone context, autophagy regulation seems to be less favorable in female than in male, leading to increased oxidative stress in female-derived OB. We recently demonstrated that autophagy deficiency in OB decreases mineralization and favors the formation of OC due to an increase in RANKL secretion, inducing an osteoporotic-like phenotype [11]. Our data suggest that autophagy regulation in female OB could, at least in part, be involved in the highest susceptibility to osteoporosis observed in women.

To determine whether OB autophagy is a critical function in female to counteract age-related bone loss in the context of estrogen fall, we performed OVX in old OB-specific autophagy-deficient or -competent mice. We first observed that OVX doesn't affect the trabecular bone volume in old control mice. Similarly, a study of Prisby et al. demonstrated that OVX had no effect on BV/TV ratio in old rats [46], suggesting that aging might be overriding the acute effects of estrogen deficiency. However, in old mice under estrogen deprivation, the loss of autophagy significantly aggravates the skeletal phenotype, suggesting that autophagy is a crucial mechanism to counteract age-related bone loss at menopause. These results are supported by the anti-aging effects of several autophagy inducers, such as rapamycin or resveratrol which are able to inhibit OC differentiation and/or activity and to prevent OVX-induced bone loss [47, 10].

Taken together, our results suggest that autophagy levels in bone cells fluctuate with age and hormonal status in a different way depending on the sex. In females, the lowering of autophagy in OB, which is associated with an increased oxidative stress, could play a role in the construction-resorption balance disequilibrium observed in osteoporosis. In addition, the loss of autophagy in OB significantly aggravates bone phenotype associated with aging and estrogen deprivation, indicating that autophagy could be a new therapeutic target in osteoporosis.

**Figure 6:**
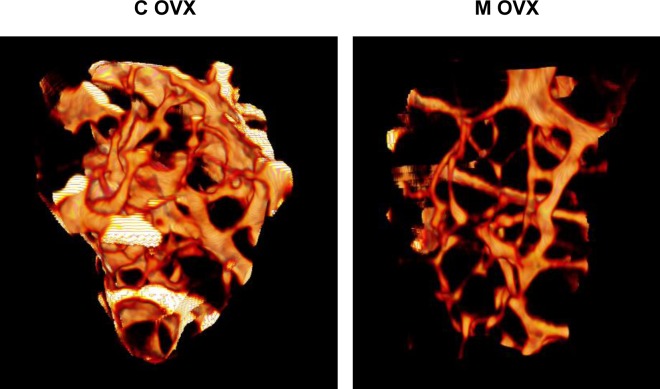
Illustration of 3D reconstructions of trabecular bone from ovariectomized control and mutant mouse using microCT 3D reconstruction of distal femur trabecular bone was performed in eighteen month-old female Atg5Col1A-Cre+ (mutant, M) mice and their control littermates (control, C) following ovariectomy. These reconstructions based on 200 section analysis, illustrate the decrease of trabecular bone volume in mutant mice.

## MATERIALS AND METHODS

### Cell culture

Primary osteoblasts were isolated from long bones according to the protocol of Bakker et al. [48]. Briefly, femur and tibia are scrapped with a scalpel, epiphyses are cutted and bone marrow was flushed out with PBS using a 5 ml syringe and a 25 gauge needle. The clean diaphyses were then cutted into 1-2 mm^2^ pieces and incubated in 4 ml collagenase II solution (2 mg/ml, Gibco, Thermo Fisher Scientific) for 2h at 37°C in a shaking water-bath to remove remaining soft tissue and adherent cells. After washing, the bone pieces were transferred in a 25 cm^2^ flask in complete medium consisting of alpha-MEM (Lonza) supplemented with 10% fetal calf serum (Hyclone, Thermo Fisher Scientific), glutamine (2mM, Sigma-Aldrich), ascorbic acid (50 μg/mL, Sigma-Aldrich). After 3 weeks of culture, cells were seeded in differentiation medium consisting of complete medium supplemented with CaCl_2_ (1.4 mM, Sigma-Aldrich) and μ-glycerophosphate (50 mg/mL, Sigma-Aldrich).

### Mice

The experiments were conducted in accordance with the French and European regulations for the care and use of research animals and were approved by the local experimentation committee (PEA n°205). *Atg5^flox/flox^ Col1-Cre+* mice were generated by intercrossing the progeny of crosses between *Atg5^flox/flox^* mice [49] obtained from the RIKEN BioResource Center, Japan (Ref RBRC 02975)I, and α*1(I)collagen-Cre* transgenic mice [50] obtained from the MMRRC (Mice ID number 208-UCD).

8-week-old male and female mice underwent gonadectomy or a sham operation under general anesthesia (100 mg/kg Ketamine combined with 10 mg/kg Xylazine). For the female mice, gonadectomy involved a small incision in both flanks after which the ovaries were removed. In the male mice, small incisions were made in the lower abdomen through which the testes were removed. Sham-operated animals underwent the same procedures without removal of ovaries or testes.

### Western blot analysis

Regarding protein extraction from cortical bone, after removing the epiphysis of both femurs and tibias and flushing the bone marrow with PBS, the bone surface was scraped with a scalpel. Bone pieces were then ground and homogenized to a fine powder in liquid nitrogen using a mixer mill (Retsh MM-400) and demineralized in EDTA. The resulting bone powder was then incubated in reducing sample buffer containing 2% SDS, 0.5% Sodium deoxycholate, 1% Igepal CA-630 (Nonidet P-40) and 0.1 M dithiothreitol for 10 min at 100°C.

Regarding cultured cells, cells were washed with phosphate-buffered saline (PBS), scraped in ice-cold PBS and centrifuged at 500 g for 5 min. The cell pellets were resuspended directly in reducing sample buffer (Laemmli : 60 mM Tris-HCl, pH 6.8, 2% sodium dodecyl sulphate (SDS), 100 mM dithiothreitol and 0.01% Bromophenol Blue) in the presence of a complete EDTA-free protease inhibitors cocktail (Roche Diagnostics). Genomic DNA was sheared by passage through a narrow-gauge syringe in order to reduce viscosity. Resulting total protein extracts were then heated at 95°C for 4 min, separated on a 14% SDS-polyacrylamide gel and electrotransferred to polyvinylidene difluoride membranes (Immobilon, Millipore). Blots were blocked for 1 h with Tris-buffered saline-0.05% Tween 20 (TBS-T) supplemented with 5% nonfat milk and incubated overnight at 4°C with primary antibodies. Filters were then washed in TBS-T, incubated for 45 min at room temperature with appropriate secondary antibodies conjugated to horseradish peroxydase and washed again prior to detection of signal with ECL plus chemilumiscent detection kit (GE Healthcare). Antibodies used in this study were mouse monoclonal anti-LC3 (clone 8E10) (MBL, Clinisciences), mouse monoclonal anti-β-actin (clone AC-15) (Sigma-Aldrich) and goat anti-mouse horseradish peroxidase-conjugated IgG (Santa Cruz Biotechnology).

### Fluorescence microscopy

For oxidative stress analysis, cells were grown in glass bottom 24-wells (PAA 21315231X) and stained with 3 μM CellROX orange reagent (Molecular probes, Life technologies C10443) and Hoechst 33342 by adding the probe to the complete medium and incubating the cells at 37°C for 30 min. The cells were washed in PBS and analyzed by fluorescence microscopy using the Microscope Axio Observer Z1 Zeiss and 2012 Zen software. For each mouse, the fluorescent cells present in 10-12 pictures at x20 magnification were analyzed using the ImageJ open source processing program, representing between 800 to 3000 cells analyzed per condition.

### Transmission electron microscopy

Cells were fixed in 1.6% glutaraldehyde in 0.1M phosphate buffer pH 7.4 immediately after medium removal or centrifugation, respectively. Samples were rinsed with the cacodylate buffer (0.1 M) and then post-fixed in osmium tetroxide (1% in cacodylate buffer) for 1 h. After rinsing with distilled water, they were then dehydrated through an increasing ethanol series and embedded in epoxy resin. Ultrathin sections (70 nm) were collected on Formvar coated copper grids, stained with uranyl acetate and lead citrate and examined with a Jeol JEM 1400 transmission electron microscope equiped with a SIS Morada Camera.

### Micro-CT analysis

Architectural parameters were analyzed by high-resolution X-ray micro-CT, using the SkyScan-1076 (SkyScan, Aartselaar, Belgium) system for small-animal imaging. Each femur was scanned parallel to its longitudinal axis (60 kV, 148 μA). A core of 200 sections, each 9 μm thick (7 mm long) was used for trabecular bone morphometry evaluations with SkyScan CtAn software. The following factors were measured: total volume, bone volume (BV) and the BV/tissue volume (TV) ratio. Trabecular BV and cortical BV were evaluated separately and the ratio of these two volumes was calculated. Trabecular bone thickness, trabecular number and separation were measured with a semi-automating morphing procedure, from total BV. Cortical thickness was evaluated on 150 sections at mid shaft of diaphysis.

### Statistical analysis

The results are expressed as mean ± SD and comparisons were performed using Student's t test. P values less than 0.05 were considered significant.

## SUPPLEMENTARY MATERIAL FIGURE


