# Supplementary material for: Sex-specific autophagy modulation in osteoblastic lineage: a critical function to counteract bone loss in female
Source: Oncotarget. 2016 Sep 13;7(41):66416–28. doi: 10.18632/oncotarget.12013 (PMC5341810; doi:10.18632/oncotarget.12013)
Supplement: Supplementary file 1 [file oncotarget-07-66416-s001.pdf]

Sex-specific autophagy modulation in osteoblastic lineage: a critical function to counteract bone loss in female

Supplementary Material

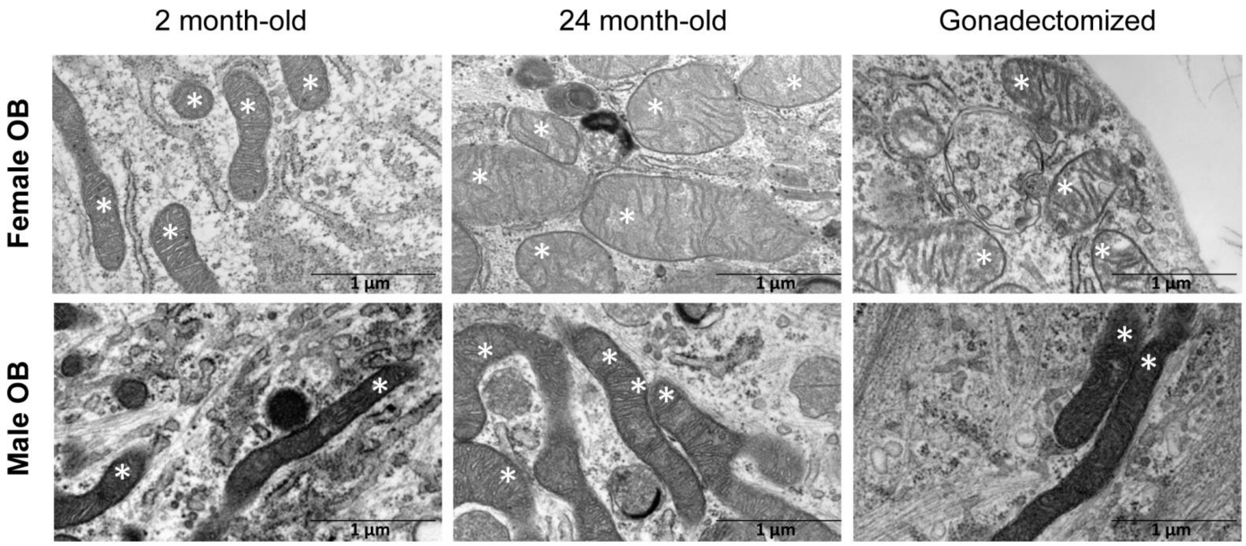

Supplementary Figure S1: Mitochondrial morphology analyzed in OB by transmission electron microscopy. Mitochondria observed in OB from 2 month-old, 24 month-old and gonadectomized male and female mice were analyzed by transmission electron microscopy. Representative micrographs are presented. White asterisk: mitochondria.
